# On the (number of) aversive traits it takes to approximate the common core of aversive personality

**DOI:** 10.1038/s41598-023-42115-z

**Published:** 2023-09-12

**Authors:** Luisa K. Horsten, Morten Moshagen, Benjamin E. Hilbig

**Affiliations:** 1 Department of Psychology, RPTU Kaiserslautern-Landau, Fortstr. 7, 76829 Landau, Germany; 2 Ulm University, Albert-Einstein-Allee 47, 89081 Ulm, Germany

**Keywords:** Psychology, Human behaviour

## Abstract

The Dark Triad and Dark Tetrad are the constellation of aversive traits that are most commonly assessed to study their common (aversive) and unique (non-aversive) features. Nonetheless, it is unclear whether these particular traits (in combination) indeed closely approximate the common core of all aversive traits. A close approximation of the aversive core, however, is crucial if one is strictly interested in an aversive trait's unique variance. Therefore, the present study aims to specify how many and which aversive traits jointly approximate the aversive core to a satisfactory extent. To this end, the aversive core was first estimated from 16 aversive traits and then correlated to the aversive cores extracted from all 63,002 possible combinations of two to eleven of these traits. Results showed that most combinations of four, or essentially any combination of at least six aversive traits, approximated the aversive core reasonably well, whereas the Dark Triad and Dark Tetrad lead to systematically poorer approximations compared to other combinations of three of four aversive traits.

## Introduction

Why do individuals differ in the tendency to intentionally behave transgressing, harmful or outright violent towards their fellow humans? From the perspective of personality psychology, socially and/or ethically aversive (“dark”) personality traits explain such differences. Aversive traits are stable personality characteristics that are linked to ethically, morally, and/or socially aversive behavior like manipulation, theft, or coercion. By definition, different aversive traits thus share a common element of social aversiveness and are, consequently, empirically substantially related to each other^1^.

Beyond what is common to all aversive traits, each aversive trait may entail unique aspects. For instance, Psychopathy is a combination of the aversive aspect of meanness—i.e., “deficient empathy, disdain for and lack of close attachments with others, rebelliousness, excitement seeking, exploitativeness, and empowerment through cruelty”^2^—and the (per se) non-aversive aspect of disinhibition—i.e., “impulse control problems entailing a lack of planfulness and foresight, impaired regulation of affect and urges, insistence on immediate gratification, and deficient behavioral restraint”^2, 3^. Given the common core of aversiveness, it is arguably the non-aversive aspects that make an aversive trait unique and distinct from other aversive traits. The distinction between aversive and non-aversive aspects thus plays an integral role in defining and studying any aversive trait.

Although there are several theoretical notions concerning the commonalities of aversive traits^4–9^, we herein focus on the conceptualization provided in the framework of the Dark Factor of Personality, D^1^, because it entails the crucial assumption that any aversive trait can be understood as a flavored manifestation of the same underlying aversive disposition. This disposition, D, is defined as the “general tendency to maximize one’s utility—disregarding, accepting, or malevolently provoking disutility for others—, accompanied by beliefs that serve as justifications”^1^ and it is assumed that D represents those aspects that make a trait aversive. As such, D is conceptualized analogously to the *g*-factor of Intelligence^10^ in that it is responsible for the shared variance among all aversive traits, whereas each aversive trait may entail additional, unique (non-aversive) aspects^3^. This conceptualization has been corroborated by several studies showing that the commonalities among different aversive traits are accounted for by a single shared factor that is in line with the definition of D^1, 11^. Research has further shown that, in turn, aversive traits have little to no predictive validity for aversive outcomes after D is accounted for^1, 12^ and that D longitudinally shapes aversive traits and socially aversive psychopathology^13, 14^, thereby lending strong support to the notion that D represents the “aversive essence” of these traits.

Nonetheless, aversive traits differ in their D saturation, i.e., in the extent to which they comprise (non-aversive) unique features beyond D. For example, some forms of Narcissism and Psychological Entitlement entail substantial unique variance beyond the scope of D, whereas other aversive traits are almost completely absorbed by D (e.g., Machiavellianism, Amoralism-Frustralia)^11, 13^. Likewise, aversive traits will vary in terms of how much they overlap conceptually with defining aspects of D. For example, Moral Disengagement almost exclusively refers to certain justifying beliefs and, thus, reflects only a relatively narrow portion of the theoretical definition of D as compared to, say, Machiavellianism which reflects justifying beliefs (e.g. cynicism, negative reciprocity, and superiority) and various forms of utility maximization at others’ expense. In summary, both in terms of variance and in terms of content, some aversive traits have relatively little in common with other aversive traits and are consequently less D saturated than other aversive traits are.

To study the impact of common (aversive) vis-à-vis unique (non-aversive) features of aversive traits, the typical approach is to investigate a set of aversive traits and partial out their common variance in explaining third variables. Thereby, one can investigate an aversive trait’s predictive validity beyond the shared aversive core^15, 16^. However, to date, the Dark Triad (i.e., Psychopathy, Machiavellianism, and Narcissism^6^) and Dark Tetrad (i.e., Dark Triad plus Sadism^17, 18^) are the constellations of aversive traits most commonly studied in this fashion. These three or four traits are commonly assessed and subsequently analysed via multivariate methods in which the regression coefficients represent the predictive validity of each predictor’s unique variance beyond the variance that is shared with the other predictors, allowing insights both into their common core and their unique features^15, 19^. As such, the underlying assumption is that whatever these three or four traits share fully represents *the* core of *all* aversive traits (i.e., D)^20^.

However, it is unclear whether these particular traits (in combination) indeed closely approximate the common core of *all* aversive traits. It has repeatedly been argued that the common element of all four Dark Tetrad traits is callous manipulation^15, 18, 19, 21^, which arguably misses out on certain commonalities of *all* aversive traits. Specifically, although callousness, i.e., a lack of empathy, is certainly an important prerequisite for aversive traits like Exploitativeness or Vengefulness, traits like Egoism or Greed are rather characterized by disregarding others’ needs. Moreover, a core of callousness would not account for certain attitudes and beliefs regarding one’s superiority or entitlement, which are prerequisites, and indeed central features, of traits like Entitlement or Moral Disengagement. Consequently, the Dark Triad/Tetrad may approximate the aversive core (and thus D) less well than other sets of aversive traits.

However, if one is strictly interested in an aversive trait’s unique variance, a close approximation of the aversive core is crucial in order to partial out any (and all) variance that the trait shares with other aversive traits. In this respect, the Dark Triad/Tetrad may thus fall short. Additionally, if one is interested in the unique variance of an aversive trait that is not included in the Dark Triad/Tetrad, it is not yet known with how many and which traits the trait of interest should be combined to partial out a close approximation of the aversive core. More generally speaking, it is an open question how many (and which) aversive traits—in combination—will provide an adequate approximation of *the* common core of *all* aversive traits, i.e. D. Indeed, D has been shown to be a fluid construct that is indifferent of the indicators and can be extracted from any sufficiently broad set of aversive traits. Specifically, it has been shown that relatively small random subsets of indicators from many aversive traits correlate almost perfectly with D measured by the full item set and yielded equivalent predictive validity^1, 13^ and an aversive trait was longitudinally predicted equally well by D modeled without the respective trait as by the trait itself^13^. However, this does not imply that any subset of aversive traits will approximate D (equally) well.

Thus, the aim of the present study is to specify (a) how many and (b) which aversive traits will jointly reflect D in its breadth to a satisfactory extent. Taking this more general approach will also shed light on how well three or four traits in general, and specifically the combination of the Dark Triad and Dark Tetrad traits approximate D. For one, it is to be expected that fewer traits will approximate D less well than larger sets; nonetheless, it is entirely open how small a set will still be sufficient. Moreover, given the varying degrees to which aversive traits are subsumed by D, certain aversive traits may be more informative (e.g., Machiavellianism, Amoralism-Frustralia) than others (e.g., Narcissistic Admiration, Moral Disengagement); which is more likely to become apparent in smaller sets). Vice versa, the more traits are combined, the less impact any one particular trait can have.

## Method

The study and analysis plan were not preregistered. Raw data, and analysis scripts are available on the Open Science Framework (OSF; https://osf.io/zaqde/?view_only=24f95805af31466783325504c9faf4e3, link blinded for peer-review). Data collection was run based on approval by the local ethics committee of the former University of Koblenz-Landau (now RPTU Kaiserslautern-Landau) and in accordance with the American Psychological Association’s Ethics Code. All participants provided informed consent and were compensated for participating.

### Procedure and measures

The study was conducted as part of the Prosocial Personality Project (PPP)^22^, a large-scale web-based study extending over several measurement occasions. Participants were recruited and rebursed via a German online panel provider (respondi). Aversive traits included in the present study were assessed at measurement occasions T3, T4, and 2020-05b. All items were answered on a five-point Likert scale (1 = “strongly disagree” to 5 = “strongly agree”). The order of scales was randomized within each measurement occasion. Moreover, at each measurement occasion, two attention check items were embedded within the scales (e.g., “Please select ‘strongly agree’ here. This serves to check your attention.”). Parts of the data reported herein have been published before in different combinations and with reference to other, independent research questions^23, 24^. A detailed documentation of the project including verbatim items of all constructs assessed, more detailed information on sample composition, sample sizes at each measurement occasion and (a priori defined) exclusion criteria, as well as a documentation of other publications based on data from the PPP is available on the OSF (https://osf.io/m2abp).

*Crudelia* was assessed at 2020-05b via the corresponding 13 items of the AMR40^11, 25^. A sample item is “I recognize only my own instincts and am driven only by my own desires”.

*Egoism* was assessed at 2020-05b via the 12-item Egoism Scale^1, 26^. A sample item is “It is hard to get ahead without cutting corners here and there”.

*Entitlement* was assessed at 2020-05b via the 9-item Psychological Entitlement Scale^1, 27^. A sample item is “I honestly feel I’m just more deserving than others”.

*Exploitativeness* was assessed at T3 via an ad-hoc translation of the 6-item Interpersonal Exploitativeness Scale^28^. A sample item is “I’m perfectly willing to profit at the expense of others”.

*Frustralia* was assessed at 2020-05b via the corresponding 14 items of the AMR40^11, 25^. A sample item is “The ends do not always justify the means” (reverse coded).

*Greed* was assessed at T3 via an ad-hoc translation of the 7-item Dispositional Greed Scale^29^. A sample item is “I can’t imagine having to many things”.

*Machiavellianism* was assessed at T3 via the corresponding 9-item scale of the German Short Dark Triad^30^. A sample item is “I like to use clever manipulation to get my way”.

*Moral Disengagment* was assessed at 2020-05b via the 8-item Propensity to Morally Disengage Scale^1, 31^. A sample item is “Some people have to be treated roughly because they lack feelings that can be hurt”.

*Narcissism* was assessed at T3 via the corresponding 9-item scale of the German Short Dark Triad^30^ (“I insist on getting the respect I deserve”) on the one hand, which captures narcissistic admiration, and the 6-item Narcissistic Admiration and Rivalry Questionnaire-Short^32^ (NARQ; “I want my rivals to fail”) on the other hand, which captures both narcissistic admiration and rivalry.

*Pathological Selfishness* was assessed at T3 via an ad-hoc translation of the corresponding 8 items of the Selfishness Questionnaire^33^. A sample item is “Sometimes you need to take advantage of other people before they take advantage of you”.

*Psychopathy* was assessed at T3 via the corresponding 9-item scale of the German Short Dark Triad^30^. A sample item is “It’s true that I can be mean to others”.

*Sadism* was assessed at T3 via the 10-item Short Sadistic Impulse Scale^1, 34^. A sample item is “Hurting people would be exciting”.

*Self-Centeredness* was assessed at 2020-05b via the corresponding 4 items of the Self-control scale^11, 35^. A sample item is “If things I do upset people, it's their problem not mine”.

*Spitefulness* was assessed at 2020-05b via the 17-item Spitefulness Scale^1, 36^. A sample item is “I would be willing to take a punch if it meant that someone I did not like would receive two punches”.

*Vengefulness* was assessed at T4 via the 10-item Vengefulness Short Scale^37^. A sample item is “I don’t just get mad. I get even”.

### Participants

The final sample for this study consisted of *N* = 1,676 participants (47% female, aged 18 to 69 years, *M* = 44.9, *SD* = 12.1, 95% native German speakers; all demographics measured at T1) who fulfilled all criteria for inclusion (described in more detail in the OSF repository for the PPP). The average lag between T3 and T4 was 85 days, the average lag between T4 and 2020-05b was 22 days.

### Analyses and results

Observed means and standard deviations for each aversive trait are available in Table S1 on the OSF.

The data were analyzed via structural equation modeling based on maximum likelihood estimation with the *lavaan* package^38^ in R^39^. To disaggregate total variance into variance common to all aversive traits and variance unique to each aversive trait, we chose a bifactor model approach. In a bifactor model, the variance that is shared among all items, i.e., their common core, is captured in the general factor, whereas remaining shared variance among subsets of items—which is beyond the common core—is captured in specific factors. In the present case, the general factor reflects the commonalities of all aversive traits, whereas the specific factors reflect the remaining shared variance among the indicators of a trait scale that is beyond D and thus unique to the respective subset^1, 40^.

As it is not possible to jointly estimate a full and a reduced version of a latent variable in a single structural equation model, factor scores were calculated for D. To this end, we first estimated the full bifactor model across all 16 traits. Specifically, D was modeled as the general factor on which the indicators of all 16 aversive trait scales loaded. Additionally, one specific factor was modeled from the indicators of each aversive trait scale. All factors were constrained to be orthogonal and were assigned a scale by fixing the loading of one indicator to 1. According to convential guidelines^41^, this model fit the data well, χ^2^(11,023) = 35,211, *p* < .001; RMSEA = 0.036, 90% CI [0.036; 0.037]; SRMR = 0.048. The factor scores from this model were retained and served as the criterion variable, that is a measure of D in its full breadth. From this model, we also extracted the explained common variance (ECV) for each trait. The ECV is the proportion of common variance in a specific factor explained by the general factor. Thus, the ECV reflects the D saturation of each trait. We provide the ECVs along with item loadings on the general and specific factors In Tables S2 and S3 on the OSF. Additionally, the fitmeasures of single factor models for each aversive trait are available in Table S4 on the OSF.

Next, we estimated a reduced D from each of the 63,002 possible combinations of two to eleven aversive traits. We stopped at eleven traits because adding any further traits hardly improved the approximation. Specifically, the median correlations of combinations of 12 traits with the core increased by less than .005 as compared to eleven traits. Analogously to the full model, reduced D was modeled as the general factor across the indicators of all included aversive traits, and specific factors were modeled for each included aversive trait. Finally, the thus obtained factor scores for D were correlated to the one obtained from the full model.

**Figure 1:**
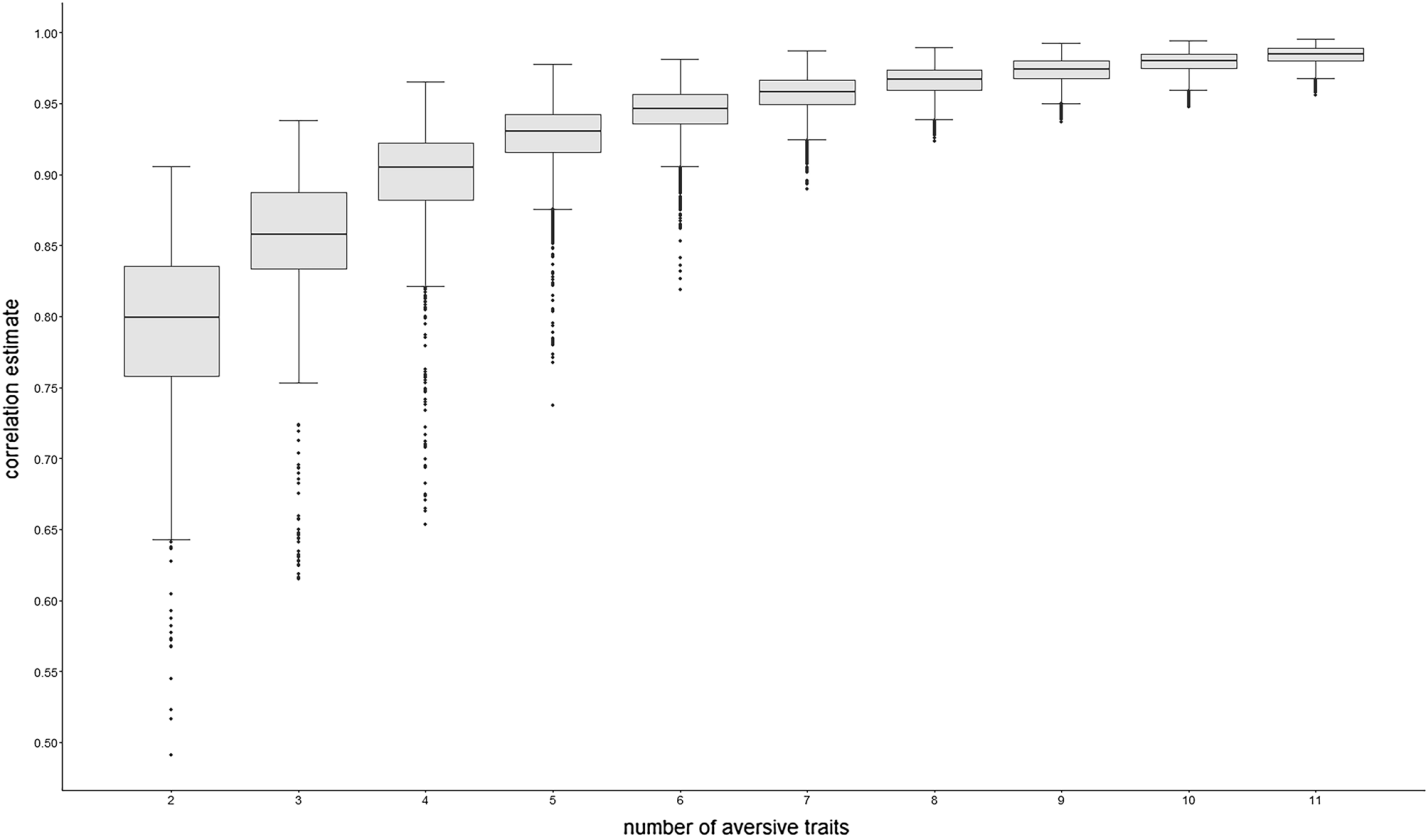
Distribution of the correlations between full D and reduced D, estimated from combinations of n aversive traits.

The median correlation of the factors scores obtained for the common core of all 63,002 combinations of aversive traits (reduced D) with the factor score obtained for the common core of the full set of aversive traits (full D) was extremely high at *r* = .96. As can be seen in Fig. 1, the median correlations between the full and reduced Ds are *r* > 0.90 for combinations of at least four aversive traits. For combinations of five aversive traits, 90% of correlations (i.e., 3,950 out of 4,389) between the full and reduced Ds were *r* > .90 (see Table 1), and from six traits onwards, the commonalities of 55,572 out of 56,134 (i.e., 99% of) combinations showed correlations of* r* > .93 with full D (median *r* = .97). Given seven or more aversive traits the approximation of D only improved negligibly for every aversive trait added.

**Table 1:** Correlations between combinations of n aversive traits and D at first, fifth and tenth percentile.

Number of traits combined	Number of combinations	percentile		
		1	5	10
2	120	.52	.57	.60
3	560	.63	.70	.79
4	1820	.72	.84	.86
5	4368	.86	.89	.90
6	8008	.90	.92	.93
7	11,440	.92	.94	.94
8	12,870	.94	.95	.95
9	11,440	.95	.96	.96
10	8008	.96	.97	.97
11	4368	.97	.97	.98

**Figure 2:**
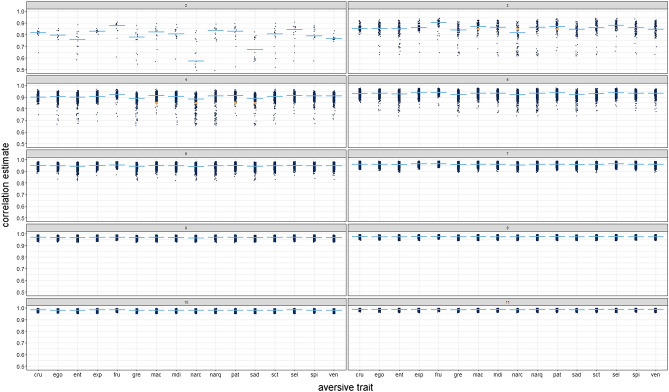
Distribution of correlations between combinations of 2–11 aversive traits including given trait and D. Yellow points in top panel in the right and second from top panel in the left column mark results of Dark Triad and Dark Tetrad combinations, respectively. *cru* Crudelia, *ego* Egoism, *ent* Psychological Entitlement, *exp* Exploitativeness, *fru* Frustralia, *gre* Greed, *mac* Machiavellianism, *mdi* Moral Disengagement, *narc* Narcissism (Short Dark Triad), *narq* Narcissism (NARQ), *pat* Psychopathy, *sct* self-centeredness, *sel* Selfishness, *spi* Spitefulness, *ven* Vengefulness.

To further understand which combinations of traits yield the better versus worse approximations of D, we examined the correlations between reduced and full D, broken down by included trait and number of traits included (Fig. 2). As expected (and indeed necessary), the more traits included, the less affected the correlations were by which traits were included. Once six or more aversive traits were included, the specific combination essentially became irrelevant. With only three or four traits included, however, combinations including Frustalia (and, to a lesser extent, Machiavellianism and Psychopathy) yielded the best approximations of full D, whereas combinations including Narcissistic Admiration (measured through the Short Dark Triad; SD3) yielded the poorest approximations. In fact, of the Dark Triad traits, only Psychopathy was part of one of the two combinations of three traits yielding the largest correlations with D (i.e., Frustralia, Self-Centeredness, and Selfishness, *r* = .94, and Frustralia, Psychopathy, and Selfishness, *r* = .94). Indeed, whereas almost all combinations of four traits yielded median correlations of *r* ≥ .90, those containing Narcissistic Admiration (median *r* = .88), Sadism (median *r* = .89), and Greed (median *r* = .89) fared slightly worse. Closer inspection of combinations including only three to six traits revealed that Narcissistic Admiration was included in all combinations that yielded the lowest 1% of correlations with full D (*r* ≤ .72, .85, and .90, respectively); Greed, Psychological Entitlement, and Sadism were also included in a majority of those combinations. Frustralia, in turn, was included in a majority of the 1% of trait combinations yielding the highest correlations with full D (*r* ≥ .95, .96, and .97, respectively).

We finally inspected how the Dark Triad and Dark Tetrad components performed in comparison to other combinations of three and four traits, respectively. Both the commonalities of the Dark Triad and the Dark Tetrad traits correlated at* r* = .85 with full D, which was lower than the median correlations of combinations of three (*r* = .86) and four (*r* = .90) traits with full D, arguably due to the inclusion of Narcissistic Admiration (as per the SD3). Compared to the median correlation of all combinations of three traits including Narcissistic Admiration (*r* = .81) with full D, the Dark Triad traits jointly performed better, but compared to the median correlations of all combinations of three traits including Machiavallianism (*r* = .87) and Psychopathy (*r* = .87), the Dark Triad traits jointly performed worse. Similarly, the Dark Tetrad traits jointly performed worse compared to the median correlations with full D that each of those traits yielded in other combination of four traits (.88 < *r* < .91). Replacing Narcissistic Admiration (SD3) with Narcissistic Admiration and Rivalry (NARQ) slightly improved approximations of full D (*r* = .88), both for the Dark Triad and the Dark Tetrad combinations. Compared to other combinations of three traits, the Dark Triad including Narcissistic Rivalry thus jointly performed slightly better, but compared to other combinations of four traits, the Dark Tetrad including Narcissistic Rivalry jointly performed slightly worse.

## Discussion

Aversive personality traits can be understood as manisfestations of a common aversive core—which, by definition, are shared across all aversive traits—along with more or less prominent unique (non-aversive) features. Indeed, the commonalities of all aversive traits can be ascribed to a common underlying disposition, the Dark Factor of Personality (D)^1^, whereas, to different extents, some aversive traits carry additional meaning beyond the scope of D^1, 2^. Correspondingly, an aversive trait’s unique features can be studied by assessing a set of aversive traits and partialing out their common variance. The constellation that is most often assessed are the Dark Triad and Dark Tetrad traits (i.e., Psychopathy, Machiavellianism, and Narcissism, along with Sadism, respectively). However, it is unclear whether these particular sets of traits actually yield a satisfactory approximation of the common core of *all* aversive traits. Especially for research questions that revolve around an aversive trait’s unique variance beyond the common core, it is, however, crucial to partial out (close to) *all* aversive variance.

Thus, drawing on about 63,000 combinations of aversive traits, the present study tested a) how many aversive traits are required and b) how useful particular traits are for a close approximation of D. Thereby, the present results help interpret (previously) published research as to how closely the common aversive core was approximated with (and, consequently, partialed out from) the respective combinations of traits assessed.

Results corroborate the fluid nature of D. Most combinations of as few as four aversive traits represented most of the variance shared by all aversive traits. With six or more aversive traits, the particular choice of traits included barely affected the meaning of their common core and from seven traits onwards additional aversive traits no longer improved the approximation of D to a relevant extent. Any combination of six aversive traits will thus yield a good approximation of the aversive core, independently of the specific choice of traits included. Nonetheless, the common core of aversive traits can be approximated reasonably well with four traits, as long as those traits are included that are most informative and those less informative are avoided.

In this regard, the present study also sheds light on how informative particular traits are for the approximation of D: the shared variances of combinations including one or more of Short Dark Triad (SD3) Narcissism, Psychological Entitlement, Greed, and Sadism were, on average, less strongly correlated with full D, whereas combinations including Frustralia were consistently superior to other combinations. These results are plausible as assessing D via traits which are relatively less subsumed by D necessarily reduces the overlap and thereby yields a slightly less adequate approximation of D. This is especially true for combinations of fewer traits in total, given the relatively higher weight any one trait has in such a scenario. Indeed, the SD3 Narcissism subscale more strongly reflects the unique variance, i.e., narcissistic admiration, and less strongly the aversive variance, i.e., narcisstic rivalry, of Narcissism^32, 42^. Greed describes a person with “an insatiable desire for more resources, monetary or other”^43^, and thus covers the aspect of utility maximization, but not necessarily at others’ costs, nor does it reference justifying beliefs. Psychological Entitlement is characterized by “a stable and pervasive sense that one deserves more and is entitled to more than others”^27^, and thus predominantly refers to justifying beliefs. These three aversive traits thus represent a comparatively narrow portion of the aspects theoretically defining D and accordingly overlap only to a more limited extent with the common core of all aversive traits. The findings are thus well in line with previous research showing that these traits are less D saturated and carry larger unique variance portions^11, 13, 44^. Sadism, on the contrary, is conceptually strongly D saturated, but (likely due to its rather extremely worded indicators) participants’ mean score on this scale was by far the lowest (*M* = 1.44, as compared to 1.75 < *M* < 2.94 for the other scales, see Table S1 in the additional materials). Such floor effects are indicative of inadequately represented true interindividual variance which, in turn, causes attenuated correlations. These results are also in line with previous research showing that neither Sadism nor Narcissistic Admiration are central in networks of aversive personality traits^44, 45^.

Vice versa, combinations including Frustralia (i.e., “resentment and a dark picture of reality which rationalize personal manipulation and Machiavellianism”^46^) were among those that showed the highest overlap with D. It thus seems to be an exceptionally informative trait which is fully in line with its conceptualization and with previous research that has shown Frustralia to be highly D saturated^11^. Frustralia is one of three facets of the Amoralism scale^25^—which aims to capture traits that prioritize selfish interests^47^—and represents amoralism caused by frustration. Accordingly, the respective items reflect beliefs and attitudes like normlessness, trust, revengefulness and consequentialism. As such, Frustralia is conceptually and operationally strongly related to justifying beliefs, which are a central aspect to D^1^. It is thus plausible that it emerged as a key trait in our results.

Interestingly, the typically investigated constellation of Psychopathy, Narcissism, and Machiavellianism lead to systematically poorer approximations of D compared to other combinations of three aversive traits. In fact, only one of the two best combinations of three traits included Psychopathy (along with Frustralia and Selfishness), whereas the other (Frustralia, Self-Centeredness, and Selfishness) does without any Dark Triad trait. The same was true for the Dark Tetrad (i.e., the Dark Triad complemented by Sadism) in comparison with other combinations of four aversive traits. Thus, although their shared variance is widely taken to represent *the* core of *all* aversive traits^6, 15, 20, 48^, they actually account for the aversive core comparatively poorly. Note that the present study allows this conclusion only for the Short Dark Triad (complemented by the Short Sadistic Impulse Scale for the Dark Tetrad). However, despite including Narcisstic Admiration (which represents more non-aversive aspects of Narcissism) this particular instrument is often recommended for its validity and brevity, and consequently one of the most widely used instruments^5, 49^. Although complementing Narcissistic Admiration by Narcissistic Rivalry (as per the NARQ) increased the Dark Triad’s correlation with full D and actually yielded a larger correlation than the median of other combinations of three aversive traits, including Narcissistic Rivalry in the Dark Tetrad still yielded smaller correlations with full D than other combinations of four aversive traits on average, which is likely due to floor effects observed in assessing the fourth trait, Sadism (see above).

In summary, the Dark Triad and Dark Tetrad traits are not unsuitable for the approximation of D, per se. Especially Machiavellianism and Psychopathy perform similarly well—if anything, even slightly better—compared to most other traits. Nonetheless, these particular combinations suffer from the inclusion of Sadism and an incomplete assessment of Narcissism as compared to other combinations of three or four aversive traits, respectively. Furthermore, results show that, in general, combinations of more traits are superior in that they capture the shared variance of all aversive traits to a fuller extent than combinations of fewer traits (like the Dark Triad and Tetrad). More generally speaking, the present results are not meant to imply that any combination of aversive traits should be substituted for a direct measure of the commonalities of aversive traits if one wants to assess the aversive core. They are rather intended as a guide to conducting and interpreting research on the unique (non-aversive) variance of aversive traits.

## Conclusion

In sum, the present study provides clear support for a fluid common disposition underlying aversive traits, that is, a common core in line with the framework of D. D, in turn, can be approximated reasonably well with most combinations of four, or essentially *any* combination of at least six aversive traits. When including only four traits, superior approximations are achieved by including traits that are strongly D saturated, like Frustralia, Machiavellianism, or Psychopathy. The shared variance of the Dark Triad and Dark Tetrad traits showed less overlap with D than other combinations of three and four traits on average and should thus not be taken as a prime representation of *the* common core of *all* aversive traits. However, if one is strictly interested in an aversive trait's unique variance, a close approximation of the aversive core is crucial. For that purpose one should thus rather assess the trait of interest along with other traits which, jointly, are able to account for partial out (close to) all aversive variance.

### Supplementary Information

Supplementary Tables.

Supplementary Table S3.

## Data Availability

Additional materials (including data and analysis scripts) are provided online on the Open Science Framework (https://osf.io/zaqde/?view_only=24f95805af31466783325504c9faf4e3, blinded for peer-review). Additional information on the Prosocial Personality Project, including verbatim items of all constructs assessed, more detailed information on sample composition, sample sizes at each measurement occasion and (a priori defined) exclusion criteria, as well as a documentation of other publications based on data from the PPP is also available on the OSF (https://osf.io/m2abp).
