# Supplementary material for: On the (number of) aversive traits it takes to approximate the common core of aversive personality
Source: Sci Rep. 2023 Sep 12;13:15021. doi: 10.1038/s41598-023-42115-z (PMC10497531; doi:10.1038/s41598-023-42115-z)
Supplement: Supplementary file 1 — Supplementary Tables. [file 41598_2023_42115_MOESM1_ESM.pdf]

Table S1
Descriptive Statistics for all assessed aversive traits

	M	*SD*
Crudelia	1.95	0.53
Egoism	2.46	0.61
Psychological Entitlement	2.70	0.67
Exploitativeness	1.75	0.68
Frustralia	2.42	0.49
Greed	2.35	0.77
Machiavellianism	2.94	0.66
Moral Disengagement	1.94	0.58
Narcissism (SD3)	2.44	0.58
Narcissism (NARQ)	2.12	0.72
Selfishness	2.13	0.70
Psychopathy	2.04	0.58
Sadism	1.44	0.48
Self-Centeredness	2.35	0.76
Spitefulness	1.78	0.51
Vengefulness	2.15	0.77

Table S2
Explained Common Variance for each specific factor

	ECV
Crudelia	.81
Egoism	.77
Psychological Entitlement	.70
Exploitativeness	.69
Frustralia	.68
Greed	.68
Machiavellianism	.62
Moral Disengagement	.61
Narcissism (SD3)	.60
Narcissism (NARQ)	.60
Selfishness	.55
Psychopathy	.48
Sadism	.42
Self-Centeredness	.40
Spitefulness	.36
Vengefulness	.23

Table S4
Single factor model fits

	χ^2	df	*p*	RMSEA	90% CI	SRMR	CFI
Crudelia	1612.631	65	.000	.119	[.114, .124]	.069	.806
Egoism	359.046	27	.000	.086	[.078, .094]	.044	.926
Psychological Entitlement	295.995	27	.000	.077	[.069, .085]	.049	.900
Exploitativeness	330.380	27	.000	.082	[.074, .090]	.050	.884
Frustralia	715.875	54	.000	.086	[.080, .091]	.052	.876
Greed	1124.694	77	.000	.090	[.085, .095]	.069	.807
Machiavellianism	243.006	14	.000	.099	[.088, .110]	.036	.951
Moral Disengagement	312.133	20	.000	.093	[.084, .103]	.048	.902
Narcissism (SD3)	338.148	27	.000	.083	[.075, .091]	.036	.948
Narcissism (NARQ)	653.186	35	.000	.103	[.096, .110]	.053	.896
Selfishness	3.830	2	.147	.023	[.000, .059]	.010	.999
Psychopathy	1217.013	119	.000	.074	[.070, .078]	.046	.895
Sadism	292.462	9	.000	.137	[.124, .151]	.059	.912
Self-Centeredness	539.629	9	.000	.188	[.174, .201]	.075	.896
Spitefulness	496.734	35	.000	.089	[.082, .096]	.031	.957
Vengefulness	266.089	20	.000	.086	[.077, .095]	.038	.949
